# A dual edge-activation strategy for rhein bilosomes enhances intradermal delivery and anti-melanoma efficacy

**DOI:** 10.3389/fphar.2026.1798385

**Published:** 2026-05-20

**Authors:** Mahmoud M. A. Ismail, Rasha M. Elkanayati, Marwa M. Essawy, Eslam Elhanafy, Ossama Y. Abdallah, Yosra S. R. Elnaggar

**Affiliations:** 1 Department of Pharmaceutics, Faculty of Pharmacy, Alexandria University, Alexandria, Egypt; 2 Department of Pharmaceutics and Drug Delivery, School of Pharmacy, The University of Mississippi, Oxford, MS, United States; 3 Department of Oral Pathology, Faculty of Dentistry, Alexandria University, Alexandria, Egypt; 4 Center of Excellence for Research in Regenerative Medicine and Applications (CERRMA), Faculty of Medicine, Alexandria University, Alexandria, Egypt; 5 Biomedical Sciences Program, Zewail City of Science and Technology, University of Science and Technology, Giza, Egypt; 6 Department of Biomolecular Sciences, School of Pharmacy, University of Mississippi, Oxford, MS, United States; 7 Department of Pharmaceutics, College of Pharmacy, Imam Abdulrahman Bin Faisal University, Dammam, Saudi Arabia

**Keywords:** bilosomes, human melanoma A375, limonene, rhein, skin cancer

## Abstract

**Introduction:**

The therapeutic potential of rhein (RH) in topical melanoma management is constrained by its poor aqueous solubility and limited intradermal bioavailability. This study proposes an innovative mechanism-driven formulation strategy to address these limitations and improve the therapeutic performance of RH against melanoma.

**Methods:**

A rhein-phospholipid complex (RH-PLC) was first prepared and and confirmed through solid-state characterization. The complex was subsequently encapsulated into two nanovesicular systems: bilosomes (BIL) engineered using sodium tauroglycocholate (STGC) as a bioactive edge activator, and limobilosomes (L-BIL), additionally containing limonene to further enhance dermal permeation. The developed formulations were evaluated for particle size, entrapment efficiency, physicochemical stability, in vitro drug release, ex vivo skin permeation, intradermal deposition, and cytotoxic activity against A375 melanoma cells.

**Results:**

Both nanovesicular systems exhibited nanoscale particle sizes (∼300 nm), high drug entrapment efficiencies (>90%), and favorable physicochemical stability. Compared with free RH, the developed systems significantly enhanced in vitro drug release, ex vivo human skin permeation (10.2-fold increase), and intradermal drug deposition (9.9-fold increase). In addition, cytotoxicity studies against A375 melanoma cells demonstrated a marked improvement in anticancer activity with IC50 values decreasing from 434.8 μg/mL for free RH to 50.4 μg/mL for BIL and 30.9 μg/mL for L-BIL. Furthermore, nanovesicular delivery improved melanoma cell selectivity and widened the therapeutic window, achieving a selectivity index (SI) of 2.4 despite the known selectivity limitations of anthraquinone derivatives.

**Conclusions:**

Rational modulation of edge-activation mechanisms can substantially improve the dermal delivery, and anticancer efficacy of RH. Among the developed systems, L-BIL demonstrated superior performance and represents a promising nanoplatform for topical melanoma therapy.

## Graphical Abstract

**Figure:**
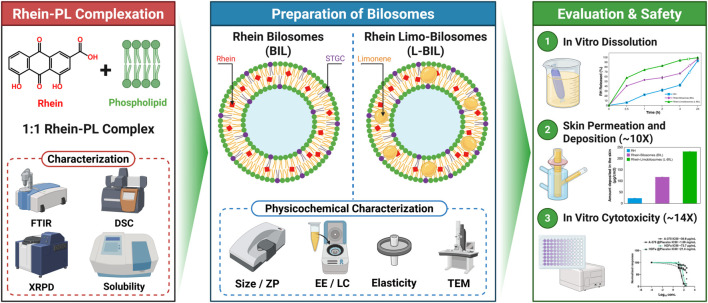
The figure was generated by BioRender.

## 1 Introduction

Topical administration represents a critically viable alternative to systemic therapy for skin cancer treatment, as it enables localized drug action at affected tissues while minimizing systemic exposure and associated adverse effects, and this approach is particularly relevant for melanoma, the most aggressive form of skin cancer, which accounts for the majority of skin cancer-related deaths worldwide (Chen et al., 2020). Melanoma originates from the abnormal proliferation of melanocytes, located at the basal layer of the epidermis, making effective drug delivery to deeper skin layers essential. However, treatment of melanoma remains challenging due to low response rates associated with existing therapies, including radiotherapy, immunotherapy, chemotherapy, and photodynamic therapy, which arise primarily from inadequate tumor targeting and the inherent resistance of melanoma cells (Ngawhirunpat et al., 2015; El-Zahaby et al., 2024).

Although localized delivery to melanocytes is essential, the stratum corneum, the outermost layer of the skin, constitutes a major physical barrier to topical drug penetration. Its highly ordered structure, with keratinized corneocytes embedded in lipid-rich intercellular regions, poses a substantial challenge for delivering compounds to deeper epidermal layers (Aziz et al., 2019). Consequently, a variety of nanoscale delivery systems have been investigated to enhance skin permeation by modulating or transiently disrupting the stratum corneum. Among these nanosystems, transferosomes, which are highly deformable vesicular carriers, have attracted considerable attention for intradermal drug delivery (Mohaddish et al., 2025). Transferosomes are composed of phospholipids combined with bioactive edge activators, which impart exceptional elasticity to the vesicle membrane, enabling deformation and passage through narrow intercellular spaces within the stratum corneum and facilitating penetration into deeper skin layers more effectively than conventional liposomes. Additionally, they may promote drug retention within the skin, thereby limiting systemic diffusion and reducing the likelihood of systemic exposure (Chen et al., 2020).

The performance of transferosome-based systems is strongly dependent on the nature of the bioactive edge activator incorporated into the vesicular membrane. Among the various classes of edge activators, bile salts have been extensively investigated due to their amphiphilic structure and surfactant properties. Phospholipid vesicles incorporating bile salts, commonly referred to as bilosomes, can impart membrane elasticity, thereby facilitating diffusion through biological barriers. Sodium tauroglycocholate (STGC) is a biocompatible bile salt that exhibits effective drug-solubilizing properties and contributes to vesicle stability by reducing aggregation during storage (Kaurav et al., 2024).

While STGC enhances skin penetration primarily through vesicle elasticization and deformability, permeation enhancement can also be achieved through a different mechanism involving direct disruption of the stratum corneum lipid architecture. Limonene, a natural monoterpene found in citrus fruits, has been widely investigated for its ability to disrupt hydrogen bonding within ceramide-rich lipid domains of the stratum corneum. Additionally, limonene has demonstrated anticancer activity by inhibiting tumor initiation and growth, suppressing angiogenesis, and promoting apoptosis in cancer cells (Wu et al., 2019; Araújo-Filho et al., 2021).

Rhein is a naturally occurring anthraquinone phytochemical derived from plant sources such as Rheum and Cassia species and was selected as the drug of choice due to its well-documented anticancer activity against melanoma. Its pharmacological effects have been attributed to mechanisms such as inhibition of cell proliferation, induction of apoptosis, and modulation of cancer-related signaling pathways. Despite these advantages, the clinical translation of RH in topical and intradermal delivery remains constrained by its poor aqueous solubility and low intradermal permeability (Wu et al., 2019; Wang et al., 2020; El-Refaie et al., 2023). These physicochemical constraints necessitate the development of advanced delivery strategies capable of simultaneously enhancing RH solubilization and penetration into deeper skin layers.

Phospholipid complexes of phytochemicals have been widely employed in topical formulations due to their ability to enhance aqueous solubility, improve biocompatibility, and integrate with skin lipids. In this study, Rhein was complexed with phospholipid to form a rhein-phospholipid complex (RH-PLC) with the aim of improving rhein solubilization and facilitating its incorporation into elastic vesicular systems. The complex was subsequently incorporated into bilosomes, in which edge activators, STGC with or without limonene, were employed to enhance vesicle deformability and support intradermal drug delivery. To further enhance skin retention and functional performance, the vesicular systems were functionalized with hyaluronic acid, a naturally occurring polysaccharide in the dermal and epidermal layers. HA serves as a gelling and stabilizing component that can prolong cutaneous retention and may promote receptor-mediated association with CD44 receptors, which are overexpressed in melanoma cells (Ji et al., 2017; Pedrosa et al., 2017; EL-Refaie et al., 2015b).

While combinations of permeation enhancers have been previously explored, their selection is often empirical and lacks systematic evaluation of individual and combined contributions. In particular, the integration of complementary enhancement mechanisms within a unified vesicular system remains underexplored. To address this gap, the present study employs a mechanism-driven dual edge-activation strategy, combining STGC-mediated vesicle deformability with limonene-induced lipid disruption. To the best of our knowledge, this specific enhancer combination has not been previously reported within a phosphatidylcholine-based vesicular system, nor applied to rhein delivery for topical melanoma treatment. Accordingly, the primary objective of this study was to design and comparatively evaluate rhein-bilosomes (BIL) containing STGC, and rhein-limobilosomes (L-BIL) incorporating both STGC and limonene as part of a continuing series of studies on rhein dermal delivery (El-Refaie et al., 2023; Ismail et al., 2025). The study systematically investigates the impact of single versus dual edge-activation strategies on physicochemical properties, drug release, intradermal permeation, and skin deposition. As a secondary objective, the optimized nanosystems were evaluated for their anticancer efficacy using the human melanoma cell line A375, alongside cytotoxicity assessment in normal human dermal fibroblasts to determine functional selectivity and therapeutic relevance.

## 2 Materials and methods

### 2.1 Materials

Rhein (RH, purity 98%) was supplied by Nutragreenlife Biotechnology Co. (Shanxi, China). Phospholipids (PL) (soy phosphatidylcholine; Lipoid® S 100) were kindly donated by Lipoid GmbH (Ludwigshafen, Germany). Limonene was gifted from Aroscent Co. (Alexandria, Egypt). STGC was obtained from Qualikems (Vadodara, India). For the cytotoxicity study, A375 human melanoma cell line was obtained from ATCC, Virginia, United States, while dimethyl sulfoxide was obtained from ThermoFisher Scientific, USA, and PBS was obtained from Biowest, USA. Hyaluronic acid was obtained from Scale Co. (Cairo, Egypt). All other reagents and chemicals were of analytical grade.

### 2.2 Preparation of rhein phospholipid complex (RH-PLC)

The rhein–phospholipid complex (RH-PLC) was prepared using the co-solvent evaporation method, following a previously reported procedure (Ebada et al., 2020). RH was dissolved in deionized water adjusted to pH 10, using aqueous ammonium solution to obtain a final concentration of 10 mg/mL. PL was separately dissolved in 20 mL of methanol. The aqueous RH solution was then combined with the methanolic phospholipid solution and magnetically stirred at 100 rpm for 1 h at room temperature to promote interaction between rhein and PL. The resulting mixture was subjected to vacuum evaporation using a rotary evaporator (Rotavapor RE-111, Switzerland) at 45 °C until complete evaporation of both solvents. The dried residue was collected and stored in a refrigerator at 4 °C until further use (Ebada et al., 2020).

### 2.3 Fourier-transform infrared spectroscopy (FTIR)

FTIR was employed to investigate molecular interactions and confirm complex formation. Approximately 5 mg of each sample (Rhein, PL, RH-PLC, and their physical mixture) was placed on the diamond crystal of the spectrophotometer (FTIR Spectrophotometer Spectrum II, PerkinElmer, USA). The samples were scanned over the wavenumber range of 450 to 4,000 cm^-1^. Spectra obtained from the Spectrum 10 software represented an average of 20 scans of 2 cm^−1^ spectral resolution.

### 2.4 Differential scanning calorimetry (DSC)

Differential scanning calorimetry (DSC) was performed to evaluate the thermal behavior of rhein, PL, and RH–PLC using a DSC 4000 instrument (DSC 4000, PerkinElmer, USA). Approximately 5 mg samples were placed in aluminum crimp cells against a reference cell and heated at a rate of 10 °C/min from 30 °C to 400 °C in a nitrogen atmosphere (20 mL/min). Data were analyzed using Pyris software.

### 2.5 X-ray powder diffraction (XRPD)

Samples of RH, PL, RH–PLC, and their corresponding physical mixture were scanned to evaluate their crystallinity using an X-ray diffractometer (XRD-D2 Phaser, Bruker, Germany) with Cu Kα1 radiation. The tube voltage and current were 30 kV and 30 mA, respectively. Diffractograms were graphed over a 2θ angle range of 1°–50° with a 0.005° step size.

### 2.6 Solubility study

RH and RH–PLC solubilities were evaluated using the shake-flask method (Torky et al., 2018). An excess of each sample was placed in a glass vial containing either deionized water or n-octanol. The vials were agitated regularly at 25 °C for 24 h. After agitation, the dispersion was allowed to stand for an additional equilibration period of 24 h to ensure that any dissolved RH or RH-PLC stabilized in the solvent. The dispersion was filtered through a 0.45 μm membrane filter to remove any undissolved particles. The concentration of dissolved rhein in the filtered solutions was measured using a spectrophotometer (Lambda 25 UV/Vis Spectrophotometer, PerkinElmer, USA) at λmax = 258 nm (Ebada et al., 2020). All measurements were performed in triplicate, and results were expressed as mean ± SD.

### 2.7 Preparation of rhein-bilosomes (BIL) and rhein-limobilosomes (L-BIL)

**FIGURE 1:**
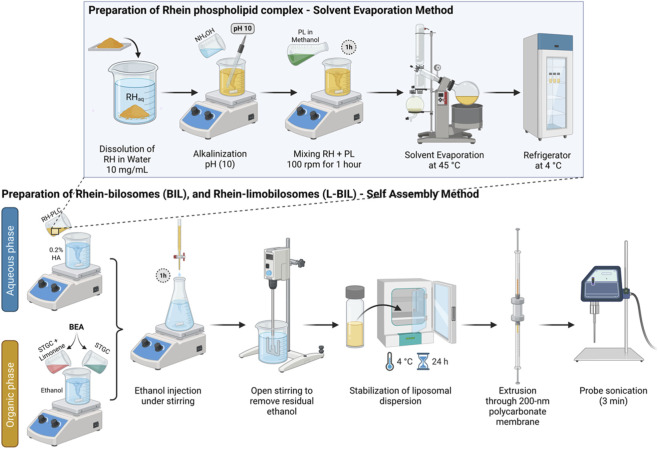
Preparation of the Rhein Phospholipid Complex (RH-PLC), Rhein-Bilosomes (BIL) and Rhein-Limobilosomes (L-BIL). The figure was generated by BioRender.

Rhein bilosomes were prepared using the ethanol injection method with slight modifications as illustrated in Figure 1. All formulations were prepared using a fixed RH: PL molar ratio of 1:1 and a constant HA concentration (0.2% w/v), while the relative amounts of STGC and limonene were systematically varied relative to the RH-PLC as illustrated in Table 1, based on ratios reported in the literature (El Maghraby et al., 2004; Rangsimawong et al., 2018; Abdelalim et al., 2020). The aqueous phase was composed of RH-PLC solubilized in deionized water containing HA and was stirred at 900 rpm using a magnetic stirrer. The organic phase, containing the edge activators limonene and/or STGC, was prepared by dissolving the components in ethanol. The organic phase was added gradually to the aqueous one under continuous magnetic stirring for 1 h at room temperature. The dispersion was then subjected to open stirring for an additional 1 h to allow removal of residual solvent (El-Zahaby et al., 2024). The resulting bilosomal dispersion was kept for 1 day in a well-closed container in the refrigerator for stabilization. Subsequently, the final formulations were extruded using an ER-1 extruder (Eastern Scientific LLC, United States) through a polycarbonate membrane (200 nm pore size, 25 mm diameter; Isopore™, Merck Millipore Ltd., Ireland) at room temperature, followed by probe sonication (Bandelin Sonoplus HD 3100, Bandelin, Germany) for 3 min to reduce particle size.

**TABLE 1:** Composition of rhein-bilosomes (BIL), rhein-limobilosomes (L-BIL), and corresponding placebo formulations.

Group	Formulation code	RH:PL	HA	STGC:PL	LM:PL	Extrusion	Sonication
Unit		Molar ratio	% w/v	w/w	w/w	Cycles	Minute
STGC- mediated elasticization (BIL)	**F1**	**1:1**	**0.2**	**1:4**	—	**5**	**3**
	F2	1:1	0.2	1:3	—	5	3
	F3	1:1	0.2	1:2	—	5	3
Dual mechanism (L-BIL)	F4	1:1	0.2	1:4	1:4	5	3
	F5	1:1	0.2	1:4	1:3	5	3
	**F6**	**1:1**	**0.2**	**1:4**	**1:2**	**5**	**3**
	F7	1:1	0.2	1:4	1:1	5	3
Placeboes	F1-P	—	0.2	1:4	—	5	3
	F4-P	—	0.2	1:4	1:4	5	3
	F5-P	—	0.2	1:4	1:3	5	3
	F6-P	—	0.2	1:4	1:2	5	3

Bold values indicate the selected optimized formulations that were advanced for further characterization and evaluation studies.

### 2.8 Physicochemical characterization of the bilosomes

#### 2.8.1 Determination of pH, particle size (PS), polydispersity index (PDI), and zeta potential

The pH of the prepared bilosomal formulations was measured using a digital pH meter (S20 SevenEasy, Mettler-Toledo, Switzerland). Measurements were performed at 25 °C, and the instrument accuracy was ±0.05 pH units. pH determination was carried out to assess the suitability of the formulations for topical application.

The average particle size (PS), polydispersity Index (PDI), and zeta potential measurements were determined using a Zetasizer (Malvern Instruments, United Kingdom) after appropriate dilution of the samples for optical clarity. All measurements were performed in triplicate, and results were expressed as mean ± SD.

#### 2.8.2 Entrapment efficiency (EE%) and loading capacity (LC%)

Entrapment efficiency (EE%) and loading capacity (LC%) were determined by separating the free drug from the vesicular dispersion using centrifugation. One mL of each formulation was centrifuged for 1 h at 10,000 rpm and 4 °C using a refrigerated ultracentrifuge (Sigma 3–30 KS, Sigma Laborzentrifugen GmbH, Germany). The amount of free rhein in the supernatant was quantified spectrophotometrically at 258 nm. EE% and LC% were determined using Equations 1, 2.

$$\text{EE}\%=\left(\text{Total drug amount}-\text{Free drug amount}\right)/\text{Total drug amount}\times 100\%$$ (1)

$$\text{LC}\%=\text{Encapsulated drug}/\left(\text{Encapsulated drug}+\text{excipients}\right)\times 100$$ (2)

#### 2.8.3 Vesicle elasticity

The vesicle’s flexibility was studied by the extrusion method (Kakkar and Kaur, 2011; Ahmed et al., 2016). Formulations were extruded using a 0.2 μm polycarbonate membrane filter. The vesicle mean size was calculated before and after extrusion using a Zetasizer (Malvern Instruments, United Kingdom) to determine the percentage change in the Z-average using Equations 3, 4.

$$\%\text{Change in vesicle size}=\left(\text{vesicle size prior to extrusion}-\text{vesicle size following extrusion}\right)/\text{vesicle size before extrusion}\times 100\%$$ (3)

$$\%\text{Change in SD}=\left(\text{Final SD }-\text{ Initial SD}\right)/\text{Initial SD}\times 100$$ (4)

#### 2.8.4 Transmission electron microscopy (TEM)

The morphology of rhein-bilosomes, with and without limonene, was examined using transmission electron microscopy (TEM). The sample was dispersed and diluted 50-fold with deionized water to ensure the particles were well-separated and could be visualized clearly. A drop of this diluted dispersion was placed on a copper grid that was pre-coated with a carbon film, to provide a stable and conductive surface for the sample. The sample was negatively stained with uranyl acetate, and excess stain was removed using filter paper. This negative staining technique enhances contrast by surrounding the nanostructures, making them easier to visualize under the TEM. After staining, the sample was air-dried to form a thin, stable film. The prepared grid was then imaged using a TEM (190 JEOL, CX model from Japan).

### 2.9 Stability studies

The developed bilosomal formulations were kept at 4 °C for 6 months and visually examined for any physical changes. Additionally, changes in Zeta potential, Z-average, and EE % were monitored throughout the storage period.

### 2.10 High performance liquid chromatography (HPLC)

A previously reported stability-indicating HPLC method was used for rhein quantification (Ebada et al., 2020). Chromatographic separation was performed on an HPLC instrument (Agilent 1,260, USA) equipped with a UV detector and a reversed-phase C18 column (25 cm, 4.6 mm, 3.5 μm particle size). The mobile phase was composed of acetonitrile and water (40:60, v/v), with the aqueous phase pH adjusted to 2.9 using orthophosphoric acid, and the mobile phase flow rate at 1.5 mL/min. The sample injection volume was 20 μL, and rhein was detected at a wavelength of 258 nm.

### 2.11 *In vitro* drug release

*In vitro* drug release was performed using the dialysis bag method. Aliquots of the formulations (1 mL, equivalent to 1 mg of rhein) were placed in dialysis bags (Visking; Serva, Germany) with a molecular weight cutoff of 12–14 kDa. The dialysis bags were submerged in 15 mL of phosphate-buffered saline (PBS) at a pH of 6.8 as the release medium. The medium was selected to mimic physiological conditions and maintain a sink condition, where RH solubility is 346.14 ± 45.94 μg/mL (Ebada et al., 2020). The setup was placed in a temperature-controlled shaking water bath at 32 °C and agitated at 100 rpm to maintain consistent mixing and diffusion of rhein from the dialysis bag into the release medium. At specific time intervals (0.25, 0.5, 1, 2, 4, and 24 h), 2 mL samples were withdrawn from the release medium and immediately replaced with fresh release medium to maintain sink condition throughout the experiment. The samples were filtered using 0.22 μm membrane filters to remove any particles and subsequently analyzed using the aforementioned HPLC method.

### 2.12 *Ex-vivo* skin deposition studies

Experiments were performed according to the OECD guidelines 428 (2004) and COLIPA guidelines (1997) (COLIPA, 1995. Guidelines for percutaneous absorption/penetration, 1997; OECD Guidelines for the testing of chemicals; skin absorption: *in vitro* method, 2011) using human skin as the gold standard model for transdermal permeation experiments (Abd et al., 2016). The protocol received approval from the Ethics Committee of the Faculty of Medicine, Alexandria University, Alexandria, Egypt (Ethical Approval No: 0,108,026). Skin samples were collected from a 30-year-old female volunteer after a cesarean section surgery. The volunteer has signed informed consent to use their samples. The permeability and skin deposition of the developed formulations were evaluated and compared with free drug suspension.

#### 2.12.1 Collection and preparation of skin samples

Normal intact skin was washed with normal saline, and the subcutaneous fat was removed. The skin was cut into pieces of about 2.5 cm^2^, wrapped in aluminum foil, and stored at −20 °C until the day of the experiment (Dreher et al., 2002).

#### 2.12.2 Permeability study

The skin was carefully mounted, with the stratum corneum facing upwards, between the donor compartment and the receptor of the Franz diffusion cell. The diffusion area was specified as 1.76 cm^2^, and the donor compartment was left open at the top to mimic the natural *in vivo* exposure of skin to air and applied substances. The Franz cells were maintained in a thermostatic shaking water bath, adjusted at 32 °C ± 0.5 °C, and agitated at 100 rpm (OECD, 2011 Guidelines for the testing of chemicals; skin absorption: *in vitro* method, 2011). This ensures consistent temperature and mixing, mimicking physiological conditions and improving diffusion. PBS at pH 6.8 was used as the receptor medium (COLIPA, 1995. Guidelines for percutaneous absorption/penetration, 1997). Prior to dosing, the skin was equilibrated with PBS for 1 h to ensure adequate hydration, after which the receptor medium was replaced with fresh PBS. Hydration ensures that the skin’s barrier properties are highly mimicking *in vivo* conditions, leading to accurate permeation results.

A 1 mL sample of each formulation, equivalent to 1 mg rhein, was applied to the donor compartment, directly on the skin surface. At predetermined time intervals (0.25, 0.5, 1, 2, 4, and 24 h), 1 mL samples were withdrawn from the receptor compartment (15 mL) to analyze the amount of permeated drug. The volume withdrawn was immediately replaced with an equal volume of fresh PBS to maintain sink conditions, keep the drug concentration gradient high and facilitate continuous diffusion. The collected samples were filtered using membrane filters (0.22 μm) to remove any particulate matter. The filtered samples were then analyzed using HPLC to determine the concentration of permeated rhein through the skin. All experiments were performed in triplicate.

#### 2.12.3 Skin retention study

At the end of the permeation experiment, the skin samples were gently removed from the Franz diffusion cells and washed using a cotton swab soaked in PBS to remove any residual drug on the skin surface, ensuring that only the drug absorbed within the skin layers would be analyzed. The cleaned skin was sliced into smaller portions and then mashed with 5 mL of methanol. The mixture was placed in a thermostatic water bath set to 37 °C and shaken at 100 rpm for 1 h to facilitate the dissolution and extraction of the drug from the skin tissues into the methanol. After incubation, the samples were sonicated for an additional 1 h. The sonication uses ultrasonic waves to disrupt the tissue matrix, aiding in the release of the drug from the skin layers into the solvent. Following sonication, the samples were centrifuged at 8,000 rpm for 20 min. The clear supernatant was collected, filtered through a 0.45-micron membrane filter, and analyzed by HPLC (Ge et al., 2014; Verma et al., 2014; Elnaggar et al., 2016)^.^ Finally, the skin retention percentage was determined according to Equation 5.

$$\text{Skin retention }\left(\%\right)=\text{Drug amount in skin}/\text{ total drug amount }X\;100\%$$ (5)

### 2.13 *In vitro* cytotoxicity and functional selectivity

The cytotoxicity of the bilosomal formulations and rhein suspension was evaluated following the MTT (3-(4,5-dimethylthiazol-2-yl)-2,5-diphenyltetrazolium bromide) assay procedures. Human melanoma cells A375 from the primary culture were trypsinized, and seeded onto plates of 96 wells with a 7 × 10^3^ density per well. Cancerous cells were treated with the proposed treatments at doses ranging from 10 to 160 μg/mL, with the equivalent volumes of their corresponding solvents. The untreated cells served as the negative control to which the metabolic activity of the treated cells was normalized.

After 48 h of incubation, cells were washed with phosphate buffer saline and incubated with MTT solution (0.05% w/v concentration in Dulbecco’s Modified Eagle Medium (DMEM), 100 µL culture media/well; SERVA Electrophoresis GmbH-purchased, Germany) followed by dimethyl sulfoxide application (100 µL DMSO/well). The indirect cell viability was estimated using an ELISA microplate reader (Infinite F15 TECAN, Switzerland) at 570 nm channel by measuring the optical density of the DMSO-dissolved formazan crystals of the treated groups relative to the optical density of untreated A375 cells.

The dose-response curves for the formulations were analyzed using GraphPad Prism software (version 8.0.1), from which the half-maximal inhibitory concentrations (IC50) were calculated.

The selectivity index (SI) for each formulation was tested using parallel MTT assays performed on normal human dermal fibroblast cells (HDFa), where SI was calculated as the ratio of the IC50 value of normal HDFa cells to the IC50 of the cancerous A375 cells.

The metabolic activity and SI of the cells were calculated according to Equations 6, 7.

$$Normalized\;response=\frac{{OD}_{treated}}{{OD}_{control}}\times 100\%$$ (6)

$$SI=IC50\;\left(HDFa\right)\;/\;IC50\;\left(A375\right)$$ (7)

### 2.14 Statistical analysis

The non-linear regression method was applied to generate the dose-dependent curves of the formulations by GraphPad Prism (version 8.0.1). The inhibition rate data was evaluated using two-way ANOVA, followed by Tukey’s multiple comparison test. Cytotoxicity assay was performed in three independent experiments, each performed in triplicate. Findings were presented as mean ± SD, and differences were considered statistically significant at p < 0.05.

## 3 Results and discussion

### 3.1 Rhein–phospholipid complex (RH–PLC) preparation and solid-state characterization

#### 3.1.1 Development of rhein–phospholipid complex

The RH–PLC was prepared using the co-solvent evaporation method, following a well-established approach reported to yield RH–PLC with high complexation efficiency (Ebada et al., 2020). Rhein exhibits enhanced solubilization under alkaline conditions due to ionization of its acidic functional groups. Accordingly, deionized water was adjusted to pH 10 using ammonium hydroxide, whose low boiling point facilitates complete removal during solvent evaporation. Methanol was chosen as the organic solvent for phospholipid dissolution owing to its excellent solvating capacity for phospholipids, miscibility with aqueous alkaline media, and ease of removal under reduced pressure. Mixing the alkaline aqueous rhein solution with the methanolic phospholipid solution produced a clear, homogeneous system, indicating favorable molecular interactions for complex formation. Subsequent solvent removal under controlled temperature resulted in the formation of a solid RH–PLC without visible phase separation, suggesting stable molecular association. A 1:1 rhein-to-phospholipid molar ratio was employed, providing high complexation efficiency.

#### 3.1.2 Fourier-transform infrared spectroscopy (FTIR) analysis

FTIR spectroscopy was employed to investigate molecular interactions between rhein and phospholipid. The FTIR spectrum of pure rhein exhibited distinct absorption bands at 3,188 and 3,061 cm^-1^, corresponding to O–H stretching vibrations of phenolic and carboxylic functional groups, a strong carbonyl stretching band at 1,695 cm^-1^ associated with the carboxylic group, and additional characteristic bands at 1,631 cm^-1^ (C=O stretching of the quinone moiety), 1,451 cm^-1^ (aromatic C=C stretching), 1,076 cm^-1^ (C–O stretching), and 748 cm^-1^ (aromatic C–H out-of-plane bending).

Upon formation of the RH–PLC, notable alterations were observed in rhein bands. The O–H stretching band at 3,061 cm^-1^ showed a marked shift toward lower wavenumbers (2,918 cm^-1^). Similarly, the quinone carbonyl band at 1,631 cm^-1^ shifted to 1,622 cm^-1^, the C–O stretching band shifted from 1,076 to 1,067 cm^-1^, while the intensity of bands at 1,451 and 748 cm^-1^ was noticeably reduced.

These spectral changes indicate that rhein participates in non-covalent intermolecular interactions through its polar functional groups. Specifically, the phenolic hydroxyl groups of rhein are likely involved in hydrogen bonding with the phosphate and ester functionalities of the phospholipid headgroup. Under alkaline conditions, partial ionization of the carboxyl group of rhein may further promote electrostatic interactions with positively polarized regions of the phospholipid head. Together, hydrogen bonding and electrostatic interactions appear to drive stable molecular association between rhein and the phospholipid supporting successful phospholipid complex formation.

#### 3.1.3 Differential scanning calorimetry (DSC)

**FIGURE 2:**
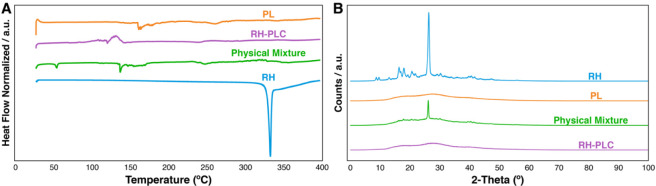
DSC thermograms **(A)** and X-ray diffractograms **(B)** of rhein, phospholipid (PL), physical mixture, and rhein-phospholipid complex (RH-PLC). The figure was generated by Microsoft Excel and retouched by Adobe Illustrator.

The thermal behavior of rhein before and after the complex formation was investigated. As shown in Figure 2A, Rhein exhibits a distinct sharp endothermic peak at 329.6 °C, corresponding to its crystalline melting point (Ascenso et al., 2015). A similar peak was observed in the physical mixture, indicating that simple blending did not alter the crystalline nature of rhein. In contrast, this endothermic event was absent in the RH-PLC thermogram, indicating loss of rhein crystallinity. This thermal behavior supports the transition of rhein to an amorphous form or its molecular dispersion within the complex, likely driven by interactions between the polar functional groups of rhein and the polar head groups of the phospholipid (Djekic et al., 2016; Freag et al., 2018; Ge et al., 2018).

#### 3.1.4 X-ray powder diffraction (XRPD)

XRPD was performed to further corroborate the DSC findings. Rhein and the physical mixture exhibited multiple sharp and well-defined diffraction peaks, including a prominent reflection at approximately 2Φ = 27°, confirming the crystalline nature of rhein. On the other hand, the RH-PLC diffractogram displayed a halo-like amorphous pattern as illustrated in Figure 2B, indicating reduced drug crystallinity and supporting its molecular dispersion within the phospholipid complex.

#### 3.1.5 Solubility enhancement of RH-PLC

The solubility assessment of RH-PLC in water and n-octanol demonstrated a notable enhancement compared to pure rhein, highlighting the amphiphilic and amorphous characteristics of RH-PLC. Rhein exhibited limited solubility in water (21.87 ± 0.02 μg/mL) and n-octanol (101.84 ± 0.13 μg/mL), consistent with its poor dissolution behavior and restricted dermal delivery. In contrast, RH-PLC showed a marked increase in aqueous solubility to (723.15 ± 3.62 μg/mL), alongside improved solubility in n-octanol (144.97 ± 0.06 μg/mL). This balanced solubility profile is particularly advantageous for dermal drug delivery, as it facilitates drug release from the formulation while promoting effective partitioning into the lipid-rich stratum corneum.

All in all, the amorphous state and enhanced solubility imparted by phospholipid complexation make RH–PLC a highly suitable precursor for vesicular encapsulation and further studies.

### 3.2 Preparation and characterization of bilosomes

The formulation strategy adopted in this study followed a two-stage, mechanism-driven approach to balance particle size, vesicle deformability, and intradermal delivery performance. Basic physicochemical properties of the bilosomal formulations, including pH, particle size, and zeta potential, were evaluated, and the values are reported in Table 2.

**TABLE 2:** Major quality attributes of rhein-loaded bilosomal formulations including Zeta-Potential, Particle Size (PS), and Entrapment Efficiency (EE%).

Formulation code	Zeta-potential	PS	PS (nm) after extrusion	Change in vesicle size after extrusion	PS after probe sonication	EE	LC	pH
	(mV)	(nm)	(nm)	%	(nm)	%	%	
**F1**	**−23.90 ± 4.56**	**1,425.00 ± 137.20**	**457.90 ± 44.86**	**67.87 ± 3.16**	**341.90 ± 97.72**	**98.77 ±0.34**	**22.78 ± 0.08**	**5.80 ± 0.07**
F2	−23.28 ± 0.65	991.00 ± 62.61	949.67 ± 98.35	4.17 ± 11.76	667.70 ± 9.48	98.42 ± 0.42	22.70 ± 0.10	5.80 ± 0.03
F3	−22.00 ± 2.57	821.30 ± 22.59	540.43 ± 10.28	34.20 ± 3.02	461.10 ± 11.62	97.45 ± 0.82	22.48 ± 0.19	6.27 ± 0.01
F4	−28.00 ± 4.95	544.00 ± 15.59	433.00 ± 90.30	20.40 ± 16.84	406.70 ± 148.20	99.18 ± 0.23	22.87 ± 0.05	5.32 ± 0.11
F5	−23.45 ± 0.85	559.93 ± 8.30	467.77 ± 10.99	16.46 ± 2.46	378.13 ± 8.55	96.51 ± 0.85	22.26 ± 0.20	5.88 ± 0.02
**F6**	**−24.43 ± 1.13**	**667.00 ± 9.71**	**609.47 ± 26.91**	**8.63 ± 4.29**	**333.53 ± 6.71**	**96.98 ± 0.14**	**22.37 ± 0.03**	**6.24 ± 0.04**
F7	−25.30 ± 0.44	650.18 ± 13.96	597.30 ± 21.66	8.13 ± 3.96	336.76 ± 6.01	99.54 ± 0.41	22.95 ± 0.09	5.65 ± 0.03

Bold values indicate the selected optimized formulations that were advanced for further characterization and evaluation studies.

In the first stage, rhein-bilosomes (BIL) were developed using STGC as the sole edge activator. STGC is known to intercalate into phospholipid bilayers, enhancing membrane fluidity and vesicle deformability. Guided by previous bilosome studies conducted by our group (Elnaggar et al., 2019; Abdelalim et al., 2020; Khalil et al., 2022; Ezzat et al., 2025), STGC:PL ratios (1:4, 1:3, and 1:2) were systematically investigated to balance vesicle deformability and structural integrity. Modulating the STGC content across formulations F1–F3 resulted in marked changes in vesicle size and extrusion-induced deformability. The initial particle sizes ranged from 821.30 ± 22.59 nm to 1,425.00 ± 137.20 nm, with higher relative STGC levels generally exhibiting reduced initial particle size, consistent with increased bilayer fluidization. Following extrusion and probe sonication, substantial size reduction was observed, particularly for F1 (STGC:PLC ratio 1:4), which achieved a mean particle size of 341.90 ± 97.72 nm. Based on its pronounced deformability and efficient post-processing size reduction, F1 was selected as the reference formulation for the second stage.

In the second stage, increasing concentrations of limonene were incorporated while maintaining a fixed STGC level. Limonene was introduced as a secondary permeation enhancer, acting primarily by modulating stratum corneum lipid domains through disruption of hydrogen bonding between ceramides (El-Zahaby et al., 2024). Rhein-limobilosomes (F4 through F7) demonstrated a marked reduction in initial vesicle sizes compared with BIL systems, accompanied by moderate deformability. Increasing limonene content resulted in further reductions of vesicle size and narrower particle size distributions following sonication indicating improved bilayer packing and size homogeneity.

The mechanisms governing transdermal drug delivery are dictated by integrated nanocarrier design parameters, including particle size, chemical composition, surface charge, lamellarity, and membrane flexibility, which collectively determine skin interaction and transport behavior (Danaei et al., 2018; Ebada et al., 2021). Following processing, the optimized formulations exhibited mean particle sizes of approximately 350 nm, a range associated with effective intradermal delivery that balances skin penetration with dermal retention while limiting systemic permeation. Submicron vesicles, particularly those incorporating penetration-enhancing components or edge activators, have been shown to access deeper skin layers compared with rigid, larger vesicles (Chen et al., 2020).

Among the evaluated systems, F6 achieved an optimal balance between particle size suitable for intradermal deposition (333.53 ± 6.71 nm), with a PDI of (0.25 ± 0.03), and adequate membrane deformability. Although F7 exhibited physicochemical characteristics comparable to F6, it was not selected for further investigation due to the use of a higher limonene content without providing a meaningful additional advantage. Consequently, F1 (BIL) and F6 (L-BIL) were selected as representative formulations for subsequent comparative evaluation of permeability enhancement and anticancer activity.

Placebo formulations (drug-free bilosomes) consistently exhibited smaller particle sizes compared to rhein-loaded systems, confirming that the observed size increase upon drug loading is expected and attributable to the incorporation of RH-PLC, which aligns with previously reported studies (El Kechai et al., 2016; Wen et al., 2021). All formulations demonstrated high entrapment efficiency (>90%), confirming that incorporation of STGC and limonene did not compromise drug association within the vesicular bilayer. The zeta potential of all formulations was consistently negative with values ranging from −22 to −28 mV, indicating sufficient colloidal stability. The pH values of the formulations ranged from 5.32 ± 0.11 to 6.27 ± 0.01, remaining within the recommended pH range for topical preparations and close to the physiological skin pH, thereby minimizing the risk of irritation (Budai et al., 2013).

In the optimized L-BIL formulation, the total amount of limonene per application was substantially below the maximum daily exposure of 10 mg/day listed in the U.S. Food and Drug Administration Inactive Ingredient Database for approved topical gel formulations. Additionally, the incorporated phospholipid and STGC levels fall within ranges previously reported for dermally approved excipients, further supporting the regulatory acceptability of the selected component concentrations. Importantly, encapsulation of limonene within nano-carriers has been shown to mitigate the direct irritant and sensitization potential of terpenes by modulating their release and reducing direct interaction with the stratum corneum (Mehanna et al., 2020). Consistent with this rationale, prior work from our research group demonstrated that repeated topical application of the rhein–phospholipid complex for 30 days did not induce visible irritation or histopathological alterations, supporting the dermal safety of the core therapeutic component (Ebada et al., 2021). Nevertheless, while the selected excipient levels remain below established regulatory exposure limits and prior laboratory findings support dermal tolerability, comprehensive repeated-dose irritation and sensitization studies evaluating the complete L-BIL formulation are warranted and will be pursued in future preclinical investigations to further confirm long-term safety (U.S. Food and Drug Administration, 2026. Inactive Ingredient Search for Approved Drug Products.; Elnaggar, 2015; El-Refaie et al., 2015a; Moustafa et al., 2017; Aziz et al., 2019; Elnaggar et al., 2019; 2025; Etman et al., 2020; Gad et al., 2024; Talaat et al., 2024; Othman et al., 2025).

**FIGURE 3:**
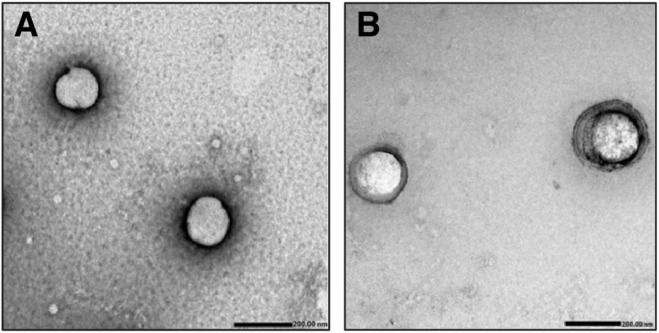
Transmission electron micrographs of **(A)** BIL and **(B)** L-BIL. Images scale bar = 200 nm.

TEM revealed that the selected formulations consisted of well-defined and spherical vesicles with smooth surfaces, as illustrated in Figure 3. BIL vesicles (F1) exhibited a unilamellar architecture, characterized by a continuous outer layer, which can be attributed to the presence of hyaluronic acid. In contrast, L-BIL vesicles (F6) displayed mixed lamellarity, with some vesicles showing multiple concentric bilayers alongside unilamellar structures. This variation in internal architecture is attributed to the incorporation of limonene, which modulates lipid packing within the membranes, without inducing vesicle disruption or structural collapse. Despite differences in lamellarity, vesicle diameters remained comparable between both formulations, in agreement with particle size measurements obtained by DLS.

### 3.3 Stability study

Stability studies were conducted to evaluate the physical stability of the selected bilosomal formulations during storage. The formulations were stored at 4 °C (±3 °C) for 6 months. Storage outside this temperature range induced irreversible physicochemical alterations. The temperature sensitivity is attributable to the thermolabile nature of constituent components, including Lipoid 100, limonene, and hyaluronic acid. Elevated temperatures can trigger multiple degradation pathways such as lipid peroxidation, hydrolytic cleavage, terpene volatilization, hyaluronic acid depolymerization, and temperature-induced colloidal phase transitions, which may compromise membrane elasticity and vesicle architecture. Throughout the storage period, no visible signs of phase separation, aggregation, or precipitation were observed, indicating satisfactory macroscopic stability. Quantitative evaluation of key physicochemical parameters was conducted and compared with freshly prepared formulations, as shown in Table 3.

**TABLE 3:** Physicochemical stability of selected bilosomal formulations during storage at 4 °C (mean ± SD, n = 3).

Formulation	Zeta potential (mV) fresh	Zeta potential (mV) 6 months	PS (nm) Fresh	PS (nm) 6 months	EE (%) Fresh	EE (%) 6 months
F1 (BIL)	−23.90 ±4.56	−12.90 ±2.29^*^	341.90 ±97.72	343.10 ±43.90 ^ns^	98.77 ±0.34	81.60 ±0.02^**^
F6 (L-BIL)	−24.43 ±1.13	−20.20 ±1.61^*^	333.53 ±6.71	230.43 ±37.09^**^	96.98 ±0.14	94.75 ±0.03^**^

*Statistical comparison was performed between freshly prepared and stored formulations using Student’s t-test.

*p < 0.05.

**p < 0.01, ns: not significant.

For particle size, both formulations remained within the nanoscale range after storage (230.43–343.10 nm). F1 (BIL) exhibited no statistically significant change (*p* > 0.05), indicating preservation of vesicle integrity. In contrast, F6 (L-BIL) showed a statistically significant reduction in particle size after storage (*p* < 0.01), suggesting improved vesicle packing and structural stabilization in the presence of limonene.

Zeta potential values were reduced over time; however, all formulations retained a moderately negative surface charge, which is sufficient to minimize aggregation during storage. In terms of entrapment efficiency, F1 (BIL) exhibited a statistically significant decrease after storage (*p* < 0.01). Similarly, F6 (L-BIL) showed a significant reduction (*p* < 0.01). Despite this decrease, both formulations maintained high levels of drug encapsulation after 6 months (>80%), particularly considering the relatively high rhein loading within the vesicular systems.

A direct comparison between BIL and L-BIL revealed that prior to storage, particle sizes were comparable with no statistically significant difference (*p* = 0.89). Following storage, L-BIL exhibited a significantly smaller particle size compared to BIL (*p* < 0.05), indicating enhanced resistance to aggregation. For entrapment efficiency, BIL initially demonstrated significantly higher values than L-BIL (*p* < 0.001); however, after storage, L-BIL maintained significantly higher drug retention compared to BIL (*p* < 0.001), highlighting the stabilizing role of limonene within the bilosomal membrane.

Overall, both formulations exhibited acceptable long-term stability under refrigerated conditions, with L-BIL demonstrating enhanced physicochemical robustness and improved drug retention, supporting the suitability of the developed nanosystems for further biological evaluation.

### 3.4 *In vitro* release study

**FIGURE 4:**
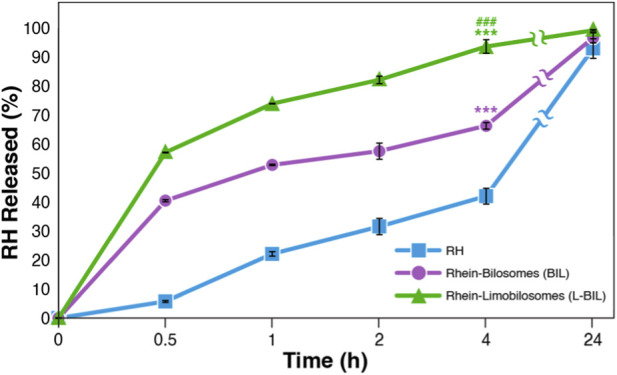
*In vitro* release profiles of rhein (RH), rhein-bilosomes (BIL), and rhein-limobilosomes (L-BIL) in a PBS release medium (pH 6.8) at 32 °C and 100 rpm. Results are expressed as mean values ± SD (n = 3). The figure was generated by Microsoft Excel and retouched by Adobe Illustrator.

*In vitro* release studies demonstrated a faster release of rhein from the vesicular formulations compared with the dissolution of the free drug from the suspension as shown in Figure 4. More than 50% of rhein was released from the nanosystems within the first hour, compared to about 20% drug dissolved from the suspension. This pronounced difference is attributed to the poor aqueous solubility of rhein and the enhanced solubilization achieved upon incorporation into the vesicular systems.

When comparing both formulations, (L-BIL) exhibited a relatively faster release profile than (BIL). This behavior may result from the incorporation of limonene, which modulates lipid packing in the vesicular bilayer and increases membrane permeability, thereby facilitating rhein diffusion into the release medium. In contrast, the bilosomal membrane of (BIL) demonstrated comparatively lower membrane permeability and a more sustained release profile. Complete release was achieved from all formulations within 24 h, as expected under the applied sink conditions. It is important to note that *in vitro* release studies are conducted in the absence of the skin barrier and therefore primarily reflect drug solubilization and carrier–drug interactions.

### 3.5 *Ex vivo* skin deposition studies

**FIGURE 5:**
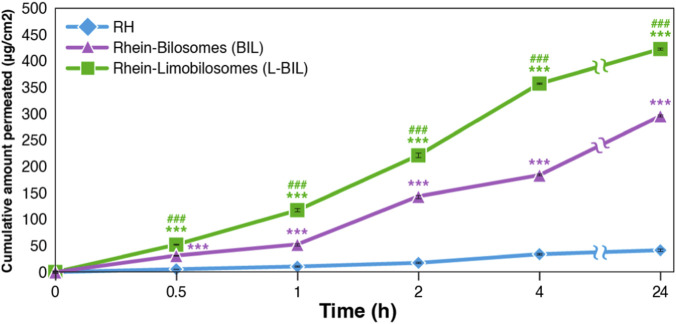
Cumulative *ex vivo* permeation of rhein (RH), rhein-bilosomes (BIL), and rhein-limobilosomes (L-BIL) through excised human skin over 24 h in PBS (pH 6.8) at 32 °C with continuous stirring (100 rpm). Results are expressed as mean ± SD (n = 3). Statistical significance was determined by two-way ANOVA with Tukey’s *post hoc* test (***p < 0.001 vs. RH; ###p < 0.001 vs. BIL). The figure was prepared in Microsoft Excel and retouched in Adobe Illustrator.

*Ex vivo* permeation and skin deposition studies were conducted using excised human skin to evaluate the potential of the developed rhein-bilosomes and rhein-limobilosomes to traverse and interact with the human skin layers. The cumulative amount of drug permeated was quantified and plotted over time, as illustrated in Figure 5. Both nanoformulations exhibited significantly higher permeation than the free rhein suspension, confirming the effectiveness of vesicular encapsulation in overcoming the stratum corneum barrier. L-BIL achieved the highest cumulative permeation, reaching approximately 423.32 ± 1.87 μg/cm^2^ (10.2 folds) over 24 h, compared 296.36 ± 2.01 μg/cm^2^ for BIL, whereas free rhein showed minimal permeation of 23.38 ± 0.03 μg/cm^2^.

In our previous study, a limonene-containing formulation demonstrated significantly greater permeation and skin deposition than BIL (*p* < 0.001), confirming the strong penetration-enhancing capability of limonene (Ismail et al., 2025). However, the dual L-BIL formulation achieved significantly higher permeation than either single-component system (p < 0.001), indicating that the combined use of limonene and STGC produces an enhanced effect beyond their individual contributions. While limonene alone favored greater superficial deposition, the dual system promoted improved overall flux across the skin barrier, suggesting a shift from predominant retention toward deeper transport.

The enhanced permeation observed for both formulations is attributed to the combined effects of their lipid-based architecture and the incorporated bioactive excipients. The phospholipid vesicular matrix promotes close interaction with skin lipids, facilitating vesicle adhesion and partitioning into deeper skin layers. STGC, a bile salt derivative, plays a key role in enhancing vesicle deformability and membrane elasticity by inserting into the phospholipid bilayer and reducing intermolecular packing forces, thereby facilitating transport through intercellular pathways. In addition, limonene acts as a terpene penetration enhancer that disrupts hydrogen bonding within ceramide-rich lipid domains of the stratum corneum, reducing barrier resistance and promoting the penetration of both hydrophilic and lipophilic agents (Abdelalim et al., 2020; Yu et al., 2024). The integration of STGC and limonene within a single nanosystem enables complementary modulation of vesicle properties and skin lipid organization, collectively contributing to the enhanced permeation observed for the L-BIL formulation.

Despite the relatively rapid initial release observed under *in vitro* sink conditions, *ex vivo* studies demonstrated sustained permeation over the 24-h period, indicating continuous delivery of rhein across the skin rather than instantaneous release. *In vitro* release experiments are conducted in the absence of the stratum corneum barrier and therefore primarily reflect enhanced drug solubilization and drug–carrier interactions rather than skin-controlled transport. Consequently, *in vitro* release data should be interpreted alongside *ex vivo* permeation and deposition results, which more accurately represent intradermal delivery behavior and are more predictive of *in vivo* performance for topical formulations.

**FIGURE 6:**
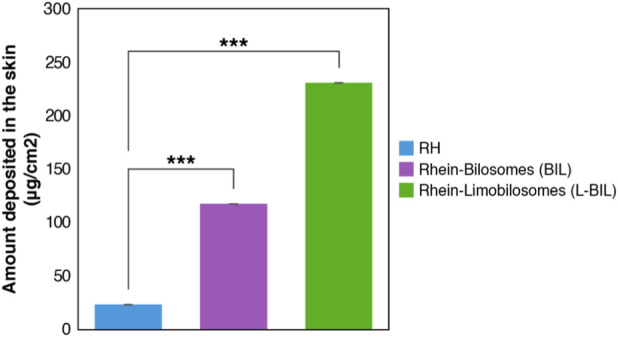
*Ex vivo* human skin deposition study of rhein, rhein bilosomes (BIL), and rhein limobilosomes (L-BIL) following 24 h permeation using excised human skin. Results are expressed as mean ± SD (n = 3). Statistical analysis was performed using one-way ANOVA followed by Tukey’s *post hoc* test. All pairwise comparisons were statistically significant (p < 0.001). The figure was generated using Microsoft Excel and retouched in Adobe Illustrator.

Following successful traversal of the skin barrier, the extent of rhein retained within the skin layers was subsequently quantified to assess intradermal deposition. Rhein-limobilosomes resulted in the highest skin deposition of 231.65 ± 4.13 μg/cm^2^ (9.9-fold) compared to 23.38 ± 0.03 μg/cm^2^ in the case of RH suspension, as illustrated in Figure 6, which can be attributed to the synergistic interactions of limonene and STGC with skin layers. Additionally, the presence of hyaluronic acid contributes to increased cutaneous retention by forming a gel-core structure, thereby prolonging residence time within the skin layers (Elhalmoushy et al., 2022).

Collectively, the *ex vivo* permeation and deposition findings demonstrate that both vesicular systems effectively enhance rhein delivery across and into the skin, with rhein-limobilosomes demonstrating superior intradermal delivery potential. Based on their favorable permeation and deposition profiles, these formulations were further evaluated for their anticancer activity against the human melanoma cell line A375.

### 3.6 *In vitro* cytotoxicity and functional selectivity

**FIGURE 7:**
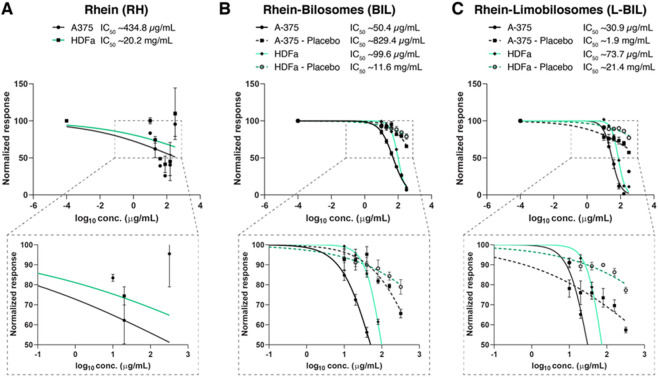
*In vitro* cytotoxicity profiles of **(A)** rhein (RH), **(B)** rhein-bilosomes (BIL), and **(C)** rhein-limobilosomes (L-BIL) against A375 melanoma cells and HDFa normal cell lines. The figure was generated by GraphPad Prism and retouched by Adobe Illustrator.

The *in vitro* anti-cancer efficacy of rhein, rhein-bilosomes (BIL), and rhein-limobilosomes (L-BIL) was evaluated against A375 human melanoma cells and compared with cytotoxicity in HDFa using the MTT assay, as demonstrated in Figure 7. Dose–response curves were generated, and IC_50_ values were calculated using nonlinear regression analysis.

Free rhein exhibited relatively weak and inconsistent cytotoxic activity against melanoma cells, with an IC_50_ of 434.8 μg/mL. The dose–response curve of rhein was characterized by a shallow slope and substantial scatter in the data, particularly at higher concentrations. This behavior reflects limited solubility and variable cellular exposure at increasing concentrations highlighting challenges in extracting robust cytotoxic parameters from the free drug.

In contrast, Encapsulation of rhein within vesicular nanocarriers resulted in a pronounced enhancement of anticancer efficacy and markedly improved dose–response behavior. Both BIL and L-BIL generated smooth, sigmoidal concentration–response curves with steeper slopes and reduced variability, indicating predictable and concentration-dependent cytotoxicity. BIL reduced the IC_50_ against A375 cells to 50.4 μg/mL (8.6-fold reduction), while further optimization through L-BIL achieved enhanced potency with an IC_50_ of 30.9 μg/mL (14.0-fold reduction).

Selectivity toward melanoma cells was assessed using the selectivity index (SI), calculated as the ratio between IC_50_ values in HDFa to A375 melanoma cells. Free rhein exhibited limited selectivity (SI = 1.3), consistent with its known non-specific cytotoxicity and narrow therapeutic window. This observation aligns with previous reports on rhein and other anthraquinone derivatives, which also exhibit elevated toxicity toward non-target cells and normal tissues (Yuan et al., 2016; Cullen et al., 2020; Wen et al., 2021; El-Refaie et al., 2023).

Notably, nanovesicular encapsulation led to a measurable improvement in functional selectivity. BIL increased the SI to 1.9, while L-BIL achieved the highest selectivity index (SI = 2.4).

SI values approaching or exceeding 2.0 are generally considered indicative of favorable anticancer selectivity, corresponding to at least a two-fold preference for cancer cells over normal cells (Oliveira et al., 2015; Bajpai et al., 2018; Laux et al., 2022). For example, a previous study evaluating cytotoxic anthraquinone derivatives in melanoma models reported SI values in the range of approximately 1.85–3.76, which were considered indicative of promising selectivity despite differences in compound structure and experimental conditions (Bajpai et al., 2018). Within this context, the selectivity achieved by L-BIL reflects a meaningful shift in the therapeutic window relative to free rhein.

Importantly, analysis of the dose–response curves reveals a clear separation between melanoma and fibroblast cytotoxicity profiles for both vesicular systems. For BIL, the concentration required to inhibit 50% of melanoma cells remained lower than that required to induce comparable toxicity in normal fibroblasts. This separation was further amplified for L-BIL, where the IC_50_ for melanoma cells (30.9 μg/mL) was substantially lower than that for fibroblasts (73.7 μg/mL), indicating a widened therapeutic window.

While absolute cancer specificity remains challenging, consistent with the known biological profile of rhein, the present nanovesicular strategy represents a meaningful advancement by enhancing anticancer potency and achieving improved functional selectivity toward melanoma cells relative to free rhein and previously reported formulations (Ismail et al., 2025). Placebo formulations showed no statistically significant cytotoxicity under the tested conditions (p > 0.05), confirming that cytotoxicity stemmed from the delivery platform rather than formulation excipients.

Importantly, when considered alongside the physicochemical data, a consistent enhancement associated with the L-BIL system becomes evident. As shown in Table 4, L-BIL exhibited significantly higher drug release within 4 h compared to BIL (p < 0.001), along with significantly greater cumulative permeation and skin deposition after 24 h (p < 0.001), indicating improved intradermal retention. This improved delivery performance translated into enhanced biological outcomes, as reflected by the lower IC_50_ and higher selectivity index observed for L-BIL relative to BIL. Taken together, these findings demonstrate that rational nanovesicular engineering enabled concurrent improvements in drug delivery, anticancer potency, and functional selectivity, with L-BIL representing a promising platform for topical melanoma treatment and a meaningful advancement over previously reported delivery strategies.

**TABLE 4:** Comparative performance of BIL and L-BIL across key functional parameters.

Parameter	BIL (F1)	L-BIL (F6)	Dual edge activation
Release (%, 4h)	66.38 ± 1.15 (95% CI: 63.5–69.2)	93.74 ± 2.33 (95% CI: 88.0–99.5)	Faster release profile
Permeation (24 h, µg/cm^2^)	296.36 ± 2.01 (95% CI: 291.4–301.3)	423.32 ± 1.87 (95% CI: 418.7–427.9)	Improved permeation
Skin deposition (µg/cm^2^)	117.95 ± 0.12 (95% CI: 117.6–118.3)	231.65 ± 4.13 (95% CI: 221.4–241.9)	Improved skin deposition
IC_50_ (µg/mL)	50.4	30.9	Reduced IC50
Selectivity index (SI)	1.9	2.4	Higher selectivity

Values are presented as mean ± SD (n = 3), with 95% confidence intervals (CI) calculated using the t-distribution. Statistical comparisons between BIL, and L-BIL, were performed using Student’s t-test for experimental parameters.

## 4 Conclusion

This study demonstrates that a mechanism-driven nanovesicular strategy can effectively overcome key physicochemical and biological limitations associated with rhein for topical melanoma therapy. Formation of the rhein–phospholipid complex successfully addressed rhein’s intrinsic solubility constraints, enabling efficient vesicular incorporation with consistently high drug entrapment efficiencies. The developed bilosomal systems exhibited suitable nanoscale size, favorable surface charge, and acceptable stability under refrigerated storage. STGC exhibited promising bioactive edge-activator properties, which were further amplified when combined with limonene. This translated into superior skin permeation and retention for L-BIL compared with BIL alone. Importantly, enhanced dermal delivery was accompanied by significant improvements in anti-melanoma efficacy reflected by substantially reduced IC_50_ values and improved dose–response consistency. Moreover, limonene incorporation further widened the therapeutic window, yielding the highest functional selectivity among the tested systems.

The present findings demonstrate promising *in vitro* and *ex vivo* performance, however, these models do not fully capture the complexity of *in vivo* melanoma. Therefore, further evaluation in melanoma-bearing animal models is required to validate therapeutic performance under physiological conditions and to investigate intradermal permeability in both diseased and healthy skin. Comprehensive biodistribution and systemic exposure assessment following topical L-BIL application is essential to confirm intradermal tumor retention, and safety relevant to clinical translation. Parallel safety studies, including skin irritation and repeated-application toxicity evaluations, are necessary to further establish tolerability. Future studies may also explore targeting strategies, including ligand-mediated recognition or stimuli-responsive release mechanisms, to further enhance melanoma selectivity.

## Data Availability

The original contributions presented in the study are included in the article/supplementary material, further inquiries can be directed to the corresponding authors.
